# Sickness absence due to common mental disorders and antidepressant prescription among health and social care workers during compared with before the COVID-19 pandemic: a nationwide register study of the Swedish population

**DOI:** 10.1093/joccuh/uiaf067

**Published:** 2025-11-25

**Authors:** Stefanie Kirchner, Katalin Gémes, Pontus Josefsson, Josep Maria Haro, Mireia Felez-Nobrega, Heidi Taipale, Marit Sijbrandij, Anke B Witteveen, Maria Melchior, Giulia Caggiu, Claudia Conflitti, Antonio Lora, Matteo Monzio Compagnoni, Jakob Bergström, Ellenor Mittendorfer-Rutz

**Affiliations:** Public Mental Health Research Unit, Department of Social and Preventive Medicine, Centre for Public Health, Medical University of Vienna, Vienna, Austria; Division of Insurance Medicine, Department of Clinical Neuroscience, Karolinska Institutet, Stockholm, Sweden; Division of Insurance Medicine, Department of Clinical Neuroscience, Karolinska Institutet, Stockholm, Sweden; Parc Sanitari Sant Joan de Deu, Institut de Recerca Sant Joan de Deu (IRSJD), Sant Boi de Llobregat, Barcelona, Spain; Parc Sanitari Sant Joan de Deu, Institut de Recerca Sant Joan de Deu (IRSJD), Sant Boi de Llobregat, Barcelona, Spain; Division of Insurance Medicine, Department of Clinical Neuroscience, Karolinska Institutet, Stockholm, Sweden; Niuvanniemi Hospital, Kuopio, Finland; Department of Clinical, Neuro- and Developmental Psychology, WHO Collaborating Center for Research and Dissemination of Psychological Interventions, Amsterdam Public Health Institute, Vrije Universiteit Amsterdam, Amsterdam, The Netherlands; Department of Clinical, Neuro- and Developmental Psychology, WHO Collaborating Center for Research and Dissemination of Psychological Interventions, Amsterdam Public Health Institute, Vrije Universiteit Amsterdam, Amsterdam, The Netherlands; Epidémiologie Sociale, Santé Mentale, Addictions (ESSMA), Institut Pierre Louis d'Epidémiologie et de Santé Publique (IPLESP), INSERM, Sorbonne Université, 75571 Paris, Cedex 12, France; Department of Mental Health and Addiction Services, Lecco, Italy; Unit of Biostatistics, Epidemiology and Public Health, Department of Statistics and Quantitative Methods, University of Milano-Bicocca, Milan, Italy; Unit of Biostatistics, Epidemiology and Public Health, Department of Statistics and Quantitative Methods, University of Milano-Bicocca, Milan, Italy; Department of Mental Health and Addiction Services, Lecco, Italy; Unit of Biostatistics, Epidemiology and Public Health, Department of Statistics and Quantitative Methods, University of Milano-Bicocca, Milan, Italy; Division of Insurance Medicine, Department of Clinical Neuroscience, Karolinska Institutet, Stockholm, Sweden; Division of Insurance Medicine, Department of Clinical Neuroscience, Karolinska Institutet, Stockholm, Sweden

**Keywords:** occupational medicine, health care workers, pandemic impact, prescription trends, workforce well-being, register-based study

## Abstract

**Objectives:**

Essential workers, particularly in health care and social services, were critical during the peak of the COVID-19 pandemic, yet their mental health outcomes remain understudied. We examined changes in (1) sickness absence (SA) due to common mental disorders (CMDs), and (2) antidepressant prescription in health and social care workers during versus pre-pandemic periods.

**Methods:**

Using Swedish national registers, we included health care and social workers (aged 19-65 years) from 2018 to 2021. We compared quarterly incidence rate (IR) trends for SA >90 days due to CMDs, and for antidepressant prescriptions, across 2 periods: pre-pandemic (January 2018 to February 2020) and during the pandemic (March 2020 to December 2021) using interrupted time-series analysis. Analyses accounted for seasonality and were stratified by age, sex, and education.

**Results:**

There was no evidence of a difference in IR trends for SA >90 days or for antidepressant prescription pre-pandemic versus during the pandemic for the entire sector. However, trends of IR for antidepressant prescription increased among workers in medical laboratories (8.7% per quarter change; 95% CI, 4.4%-13.1%) and hospitals (1.5%; 95% CI, 0.6%-2.5%) and decreased per quarter for ambulance transports (5.4%; 95% CI, 0.4-10.0%). Women (10.9%; 95% CI, 7.2%-14.7%) and highly educated individuals (10.0%; 95% CI, 4.1%-16.1%) working in medical laboratories as well as 19-25-year-olds working in primary and dental care (7.3%; 95% CI, 1.7%-13.1%) also experienced an increase in antidepressant prescription.

**Conclusions:**

Although overall trends in SA >90 days and in antidepressant prescription remained stable, certain occupational and sociodemographic groups were found to be affected in regard to antidepressant prescription. These groups warrant targeted support in future health crises.

## Teaser

**Key points**

*What is already known on this topic?*

Before the COVID-19 pandemic, health care and social workers in Sweden already showed higher levels of antidepressant use and sickness absence due to mental disorders compared with other professions. The pandemic may have worsened both their physical and mental health. Prior research focused mainly on short-term effects using self-reported data. However, long-term sickness absence is linked to lasting employment challenges. By using sickness absence and antidepressant prescription as outcomes, both the social consequences of mental disorders and the effects of evolving changes in the work environments can be captured. Understanding variations between occupational subgroups is essential for tailoring support strategies in future health crises.

*What this study adds?*

This study found no overall shift in mental health trends across the entire health care and social service workforce, but notable differences emerged among subgroups. Workers in hospitals and medical laboratories saw increased antidepressant use during the pandemic. These increases were especially pronounced among younger hospital staff, and among women and those with higher education in medical labs.

*How this study might affect research, practice, or policy?*

The findings highlight the uneven impact of the pandemic on mental health across health care roles. This emphasizes the need to consider subgroup-specific risks in policy planning and crisis response. Even before the pandemic, mental health challenges and work strain were rising in some sectors, such as ambulatory settings. Ongoing monitoring of mental health outcomes and work conditions remains critical.

## 1. Introduction

The COVID-19 pandemic posed an unprecedented global challenge for public health, with several implications for the population’s health.^1,2^ Health care and social workers emerged as a focal point of attention, being regarded as particularly vulnerable given their frontline roles during the pandemic and the potential impact on their physical and mental health. Prior to the COVID-19 pandemic this group of workers already had higher levels of antidepressant use and sickness absence (SA) due to mental disorders compared with individuals working outside human services such as people working in offices.^3,4^

So far, only 1 study has investigated SA due to mental ill-health in health care workers during the first year of the COVID-19 pandemic. This observed a spike in incident short-term SA (ie, ≤28 days) between March and April 2020, which diminished by May/June 2020.^5^ Although this suggests an increase in acute stress-related disorders, there are no studies available that examined longer-term effects of the pandemic on mental ill-health, that is, long SA spells representing the more severe cases. This is important, as extended periods of SA hinder future employment opportunities and lead to lasting challenges in the job market.^6,7^ Furthermore, most studies focused only on a short observation period either at the start of the pandemic or in between; studies with an observation period longer than a few months are needed to better understand the extent of the problem.^5,8,9^

Most of the studies have also relied not on health registers but on self-reported assessments in smaller populations, which can introduce recall bias and certain response patterns. Research using objective outcomes such as common mental disorders (CMDs) diagnosed by physicians or prescribed antidepressants still remains limited. By adding data on SA as an outcome measure, however, both the social consequences of CMDs and the effects of evolving changes in the work environments can be captured.^10^

It is also important to examine differences between specific subgroups. For many workers in the health care and social sector, working conditions during the pandemic worsened, typified by long work hours, cancelled holidays, and understaffed workplaces; these adverse physical and psychosocial working conditions resulted in a high psychological and emotional demand and low control.^11,12^ These changes impact the occupational groups within this sector differently, therefore the impact on mental health is also different. For example, in previous studies,^8,9,13^ technical staff working in health care, paramedics and nurses showed increased signs of distress compared with physicians.

In addition to this, sociodemographic characteristics also play a role, possibly resulting in varying impacts of the pandemic on mental health. For example, studies reported worsened mental ill-health for females or people with a low educational level during the pandemic as compared with males or people with a high educational level.^5,8,14-17^ Age was also found to modify the effect of the pandemic on mental health, although the findings are inconclusive.^5,14^ Examining the differences between the subgroups is crucial in order to design tailor-made support strategies for specific groups in case of future health crises.

Given these premises, we carried out a nationwide register study using an interrupted time-series design based on Swedish registers, aimed at: (1) comparing trends in the incidence of SA >90 net days attributed to CMDs and of antidepressant prescriptions among health care professionals and social workers in Sweden during compared with before the COVID-19 pandemic; and (2) assessing whether changes in incidence rates (IRs) of SA and antidepressant prescription differed by age, sex, or educational level.

## 2. Methods

This was an interrupted time-series design study based on Swedish register data.

### 2.1. Register data

Data were retrieved from individually deidentified linked register data of Sweden, in particular from the following administrative nationwide registers:

- LISA—Longitudinal Integrated Database for Health Insurance and Labor Market Studies (Statistics Sweden): this register provides data on sociodemographic characteristics such as sex, age, educational level, country of birth, family situation, current place of living, and unemployment^18^
- MIDAS—Micro Data for Analysis of the Social Insurance database (Social Insurance Agency): provides data on labor market–related factors such as the length and grade of diagnosis, specific disability pension, and SA^19^
- NPR—National Patient Register: provides details on specialized in- and outpatient care including date of stay and diagnoses^20^
- PDR—Prescribed Drug Register (National Board of Health and Welfare): medication that was prescribed using Anatomical Therapeutic Chemical Classification (ATC) codes, the date, and defined daily doses (DDDs).^21^

### 2.2. Study population

We included Swedish residents who were aged 19-64, working in the health care or social sector, and had lived in Sweden in the year before the start of the quarter of the observation period (ie, who were registered in LISA on December 31, 2018, 2019, 2020, and 2021). We followed the study populations by using quarterly observations of the outcomes from the beginning of the observation period starting in the first quarter of 2018 and ending the observation period in the fourth quarter of 2021. In the analyses involving SA as the outcome measure, individuals with a disability pension at baseline (ie, the beginning of the first quarter of the observation period) were excluded. Further details on the use of Swedish register data to examine IRs of mental health outcomes during compared with pre-pandemic periods have been reported elsewhere.^22^

### 2.3. Occupational groups

Individuals in gainful employment (either receiving an income or benefits related to work as registered in LISA) were categorized into occupational groups for each year before the observation year using the Swedish Standard Industrial Classification (SNI), which is based on NACE Rev. 2—the European classification of economic activities.^23^ We only included individuals who were working in the health care or social sector, that is, who were working in hospitals, health care facilities, ambulance services, or care facilities such as retirement homes.

Our study population consisted of the following large groups: “Hospitals,” “Primary and dental care,” “Primary care, not physicians,” “Residential care activities, except elderly care,” and “Social work.” Some of the smaller occupational groups were merged into one of the larger categories (see Table S1). Occupational groups covering medical laboratories, ambulance transports, or care in special forms of accommodation for the elderly were kept as separate categories, as we wanted to retain information about groups that might have been particularly affected by the pandemic.

### 2.4. Outcome measures

We used 2 outcome variables for our study: SA >90 net days due to CMDs, and antidepressant prescriptions. In Sweden, all residents aged 16-65 who receive income either from work or from parental, student, or unemployment benefits, are eligible for sickness benefits from the Social Insurance Agency (SIA), if they are unable to work due to illness or injury. For employed individuals, the first 14 days of SA are usually covered by the employer. We defined our outcome as SA for more than 90 net days due to a CMD as an incident SA spell with a main diagnosis of depressive, stress-related, or neurotic disorder identified by the International Classification of Diseases (ICD-10) codes as F32-F34, F40-44, and F48. We used a cut-off point of >90 days, as in Sweden after 90 days of SA an assessment is carried out on whether the employee is able to perform work duties other than the ones they were originally assigned to with their employer.^24^

The second outcome, antidepressant prescription, was defined as at least 1 first purchase (ie, excluding those with any prior prescriptions) of the prescribed substance during the quarter using the ATC code N06A (WHO Collaborating Centre for Drug Statistics Methodology, 2023).

The outcome measures were measured at the beginning of each quarter of the observation period (ie, every discrete incident SA spell exceeding 90 days for SA >90 days due to CMD, and every first purchase of a prescribed substance for antidepressant prescription).

### 2.5. Covariates

Sociodemographic variables such as sex (women, men) and country of birth (Swedish-born, foreign-born) were assessed on December 31, 2017. Other covariates were measured at the end of the year for each year during the observation period (ie, 2017, 2018, 2019, and 2020): such as age, categorized into groups (19-25, 26-35, 36-45, 46-55, 56-64 years); education level (elementary ≤9 years, high school 10-12 years, university/college >12 years, any missing information is included in “elementary”); family situation (married/cohabitant without children living at home, married/cohabitant with children living at home, single without children living at home, single with children living at home; children living at home); area of living (cities, towns and suburbs, rural areas); net days of SA during the year (0, 0-90, 91-180, 181-365) as well as disability pension measured at the end of the year preceding the observation year (yes, no).

### 2.6. Data analysis

We used an interrupted time-series design, using the World Health Organization’s declaration of the start of the COVID-19 pandemic on March 11, 2020 as the point in which the time series was interrupted (exposure) and the pandemic began (quarter 2 of 2020 in our dataset; 2020Q2) to examine differences in trends of the outcome variables across the occupational groups comparing the time before versus during the pandemic.^25^ For computational purposes, the data were aggregated by quarters and occupational groups into all individuals experiencing either one of the outcomes (ie, SA >90 days due to CMD or antidepressant prescription). We chose an interrupted time-series design as opposed to a cohort study design as the latter might not be as effective in capturing the immediate and evolving impact of the pandemic.

We estimated linear trends in IRs, along with 95% CIs, using log-linear Poisson regression. To assess the quarterly percentage change in IR trends before and during the pandemic, we calculated incidence rate ratios (IRRs) by dividing the IR trend during the pandemic by the IR trend pre-pandemic, with corresponding 95% CIs. Each occupational group was analyzed in a separate model. To account for persons occurring in more than 1 quarter, SEs from the general linear models were adjusted using a sandwich estimator. We used 2 time variables to enable an analysis of a change in direction of the linear trend at the interruption, that is, at the start of the pandemic (ie, 2020Q2). Of these 2 time variables, one describes all quarters of the observation period to give a trend estimate. The other time variable comprises the quarters from the start of the pandemic until the end of the observation period (ie, 2020Q2-2021Q4).

Given the seasonal variation in the outcomes, an indicator variable was introduced for each quarter of the year into the model. The quasi-likelihood information criterion (QIC) was employed to test the fit of the model with a seasonality component as compared without. As the model with adjustment for seasonal variability showed a better fit, we kept this adjustment in our analyses. We added the total number of days each individual contributed in each quarter as an offset in the model to accommodate different follow-up durations, for example, due to migration or death.

Statistical analyses were also stratified by sex, age, and education to scrutinize variations in IRs between pre-pandemic and pandemic periods within the occupational groups. In the stratified analyses, we introduced an interaction term involving pre- and pandemic periods (the exposure) and sex, age, or education (our effect modifier) into the model. We used R v.4.1.3 for the analyses and STATA v.17 for aggregating the data.

## 3. Results

The general descriptive characteristics of the study population (*n* = 757 018) are shown in Table 1. There were more females working in health care or social services than males (*n* = 605 482; 80.0%). The overwhelming proportion of individuals were born in Sweden (78%). With regard to the specific occupational groups, most individuals worked in social work (*n* = 229 209; 30.3%), followed by hospitals (*n* = 190 450; 25.2%) and elderly care (*n* = 142 255; 18.8%).

**Table 1:** Sociodemographics of the Swedish population under study (measured before the start of observation period).

**Characteristics**	**Total, *n* (%) (*N* = 757 018)**
**Sex**	
**Women**	605 482 (79.98)
**Men**	151 536 (20.02)
**Age group, y**	
**19-25**	69 744 (9.21)
**26-35**	167 819 (22.17)
**36-45**	164 796 (21.77)
**46-55**	191 199 (25.26)
**56-64**	163 460 (21.59)
**Level of education** ^**a**^	
**Elementary (≤9 y)**	52 417 (6.92)
**High school (10-12 y)**	352 669 (46.59)
**University/college (>12 y)**	351 932 (46.49)
**Family situation** ^**a,b**^	
**Married or cohabitant without children**	160 696 (21.23)
**Married or cohabitant with children**	245 269 (32.40)
**Single without children**	294 924 (38.96)
**Single with children**	56 129 (7.41)
**Area of living** ^**a**^	
**Cities**	281 494 (37.18)
**Towns and suburbs**	324 408 (42.85)
**Rural areas**	151 116 (19.96)
**Country of birth**	
**Sweden**	591 239 (78.10)
**Other**	165 779 (21.90)
**Any disability pension** ^**a**^	
**No**	743 941 (98.27)
**Yes**	13 077 (1.73)
**Sick absence days** ^**c**^	
**None**	632 181 (83.51)
**1-90**	92 457 (12.21)
**91-180**	18 797 (2.48)
**181-365**	13 583 (1.79)
**Occupational group**	
**Hospital**	190 450 (25.16)
**Primary and dental care**	81 035 (10.70)
**Medical laboratories**	3038 (0.40)
**Ambulance transports**	3860 (0.51)
**Primary care, not physicians**	18 724 (2.47)
**Residential care activities, except elderly care**	88 447 (11.68)
**Elderly care**	142 255 (18.79)
**Social work**	229 209 (30.28)

^a^Measured at the end of the year preceding the observation year (ie, 2017, 2018, 2019, and 2020). ^b^Children living at home. ^c^Measured during the year preceding the observation year.

### 3.1. Main analyses

**Figure 1:**
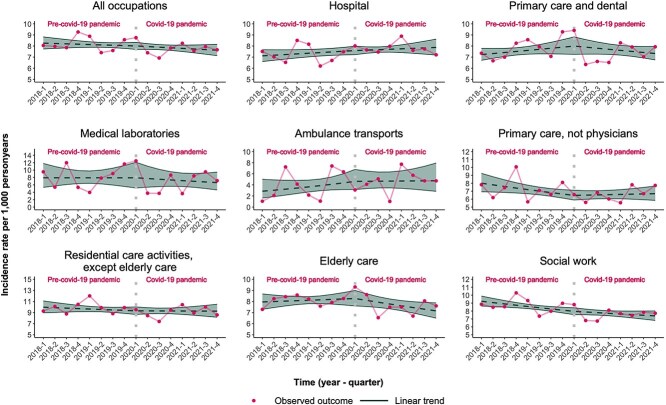
Incidence rates (IRs) of long-term sickness absence (SA) due to common mental disorders (CMDs) per 1000 person-years for health care professionals in the time pre-pandemic (ie, preceding the start of the COVID-19 pandemic, starting with the first quarter of 2018) and after the onset of the pandemic (starting from the second quarter of 2020 until the fourth quarter of 2021), adjusted for seasonality.

**Figure 2:**
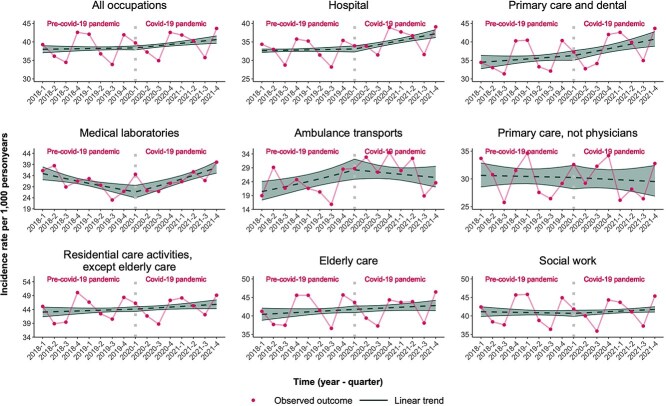
Incidence rates of antidepressant prescriptions per 1000 person-years among health care professionals in the time pre-pandemic (ie, preceding the start of the COVID-19 pandemic, starting with the first quarter of 2018) and after the onset of the pandemic (starting from the second quarter of 2020 until the fourth quarter of 2021), adjusted for seasonality.

Figure 1 shows the IRs and 95% CIs for SA >90 days due to CMDs, and Figure 2 the IRs of antidepressant prescriptions for all health care professionals pooled as well as stratified for the different subgroups. For SA >90 days due to CMDs the observed IR was 8.3 (95% CI, 7.7-8.8) per 1000 person-years in the first quarter of 2018; this remained largely unchanged until the first quarter of 2020 (8.0; 95% CI, 7.7-8.3) and slightly decreased to 7.6 (95% CI, 7.1-8.2) in the fourth quarter of 2021. In the first quarter of the study period (2018Q1), SAs >90 days were highest for people working in residential care (excluding elderly care; IR: 10.0 per 1000 person-years; 95% CI, 9.0-11.1) and social work (IR: 9.2 per 1000 person-years; 95% CI, 8.6-9.9).

For antidepressant prescription, the observed IR was 38.0 (95% CI, 37.1-39.0) per 1000 person-years in 2018Q1, stayed at about the same level in 2020Q1 (38.4; 95% CI, 37.9-38.9) and increased to 40.3 (95% CI, 39.6-41.6) in 2021Q4. Like long-term SA due to CMDs, IRs for antidepressant prescription were highest in 2018Q1 for people working in residential care (excluding elderly care; 43.1 per 1000 person-years; 95% CI, 41.4-44.8) and social work (41.1; 95% CI, 39.9-42.5). A constant increase in IRs of antidepressant prescription could be observed for physicians working in primary or dental care [2018Q1 IR: 34.5 per 1000 person-years (95% CI, 32.8-36.3); 2020Q1 IR: 36.3 (95% CI, 35.0-37.6); 2021Q4 IR: 40.8 (95% CI, (38.9-42.8)] as well as people working in residential care [2018Q1 IR: 43.1 per 1000 person-years (95% CI, 41.4-44.8); 2020Q1 44.1 (95% CI, 43.3-45.0); 2021Q4 IR: 45.8 (95% CI, 44.3-47.4)] or elderly care [2018Q1 IR: 40.4 per 1000 person-years (95% CI, 38.8-42.1); 2020Q1 41.8 (95% CI, 40.9-42.7); 2021Q4 IR: 42.8 (95% CI, 41.5-44.2)] (Figure 2).

Overall, however, there was no evidence of a difference in IR trends for either outcome measure for all occupational groups combined pre-pandemic versus during the pandemic (Tables 2 and 3). With regard to the different subgroups, no change in trends of IR of SA >90 days due to CMDs in the time during the COVID-19 pandemic as compared with pre-pandemic could be observed (Table 2).

**Table 2:** Change in slope incidence rates and slope incidence rate ratios (during vs before COVID-19 pandemic) of long-term sickness absence due to common mental disorders per 1000 person-years among health care professionals in the time pre-pandemic (ie, preceding the start of the COVID-19 pandemic, starting with the first quarter of 2018) and after the onset of the pandemic (starting from the second quarter of 2020 until the fourth quarter of 2021), adjusted for seasonality.

	**Before COVID-19 pandemic**		**During COVID-19 pandemic**		**During vs before COVID-19 pandemic**	
**Occupational groups**	**Slope IR (95% CI)**	***P* value**	**Slope IR (95% CI)**	***P* value**	**Slope IRR (95% CI)**	***P* value**
**All occupations**	0.996 (0.985-1.007)	.496	0.993 (0.981-1.006)	.275	0.997 (0.977-1.018)	.770
**Hospital**	1.008 (0.997-1.019)	.155	1.005 (0.988-1.023)	.568	0.997 (0.973-1.022)	.815
**Primary care and dental**	1.013 (0.994-1.032)	.183	0.988 (0.968-1.007)	.213	0.975 (0.941-1.010)	.166
**Medical laboratories**	1.000 (0.915-1.093)	.997	0.975 (0.895-1.061)	.555	0.975 (0.832-1.143)	.753
**Ambulance transports**	1.062 (0.966-1.168)	.215	1.004 (0.914-1.102)	.941	0.945 (0.806-1.107)	.483
**Primary care, not physicians**	0.974 (0.951-0.997)	.029	1.005 (0.982-1.028)	.695	1.032 (0.993-1.072)	.112
**Residential care activities, except elderly care**	0.992 (0.973-1.011)	.395	0.999 (0.976-1.023)	.939	1.008 (0.970-1.047)	.703
**Elderly care**	1.005 (0.986-1.024)	.622	0.980 (0.960-0.999)	.040	0.975 (0.941-1.010)	.158
**Social work**	0.982 (0.969-0.995)	.006	0.989 (0.974-1.005)	.173	1.008 (0.983-1.034)	.538

Abbreviations: IR, incidence rate; IRR, incidence rate ratio.

**Table 3:** Change in slope incidence rates and slope incidence rate ratios (during vs before COVID-19 pandemic) of antidepressant prescriptions per 1000 person-years among health care professionals in the time pre-pandemic (ie, preceding the start of the COVID-19 pandemic, starting with the first quarter of 2018) and after the onset of the pandemic (starting from the second quarter of 2020 until the fourth quarter of 2021), adjusted for seasonality.

	**Before COVID-19 pandemic**		**During COVID-19 pandemic**		**During vs before COVID-19 pandemic**	
**Occupational groups**	**Slope IR (95% CI)**	***P* value**	**Slope IR (95% CI)**	***P* value**	**Slope IRR (95% CI)**	***P* value**
**All occupations**	1.001 (0.997-1.005)	.547	1.008 (1.004-1.013)	<.001	1.007 (1.000-1.014)	.058
**Hospital**	1.002 (0.998-1.006)	.370	1.017 (1.011-1.023)	<.001	1.015 (1.006-1.025)	.002
**Primary care and dental**	1.006 (0.997-1.016)	.186	1.017 (1.007-1.026)	.001	1.010 (0.993-1.027)	.243
**Medical laboratories**	0.967 (0.946-0.988)	.002	1.051 (1.029-1.072)	<.001	1.087 (1.044-1.131)	<.001
**Ambulance transports**	1.041 (1.014-1.069)	.003	0.986 (0.955-1.017)	.366	0.946 (0.900-0.996)	.033
**Primary care, not physicians**	0.998 (0.985-1.012)	.824	0.997 (0.980-1.014)	.688	0.998 (0.973-1.024)	.885
**Residential care activities, except elderly care**	1.003 (0.997-1.009)	.320	1.005 (1.000-1.011)	.070	1.002 (0.993-1.012)	.621
**Elderly care**	1.004 (0.998-1.011)	.209	1.004 (0.998-1.009)	.222	0.999 (0.989-1.009)	.906
**Social work**	0.999 (0.993-1.004)	.618	1.004 (0.999-1.008)	.086	1.005 (0.996-1.014)	.249

Abbreviations: IR, incidence rate; IRR, incidence rate ratio.

Regarding the prescription of antidepressants, the trends in IR changed quarterly, with 8.7% during the pandemic for people working in medical laboratories (95% CI, 4.4%-13.1%; *P* < .001), and 1.5% for people working in hospitals (95% CI, 0.6%-2.5%; *P* = .002) compared with trends before the pandemic. Trends in IR decreased by 5.4% for people working in ambulance transports (95% CI, 0.4%-10.0%; *P* = .033; Table 3) during the pandemic as compared with pre-pandemic.

### 3.2. Stratified analyses

For incident antidepressant prescription, trends of IR increased for people aged 19-25 years working in primary and dental care (7.3%; 95% CI, 1.7%-13.1%) as well as in hospitals (5.4%; 95% CI, 2.9%-8.0%) during the pandemic compared with the time pre-pandemic (see Table S2 and Figure S1). Trends of IR for antidepressant prescription furthermore increased for women (10.9%; 95% CI, 7.2%-14.7%) and for people with a university or college degree (10.0%; 95% CI, 4.1%-16.1%) working in medical laboratories (see Tables S3 and S4, and Figures S2 and S3, respectively).

## 4. Discussion

The study investigated the effect of the COVID-19 pandemic on antidepressant prescriptions and on long-term SA due to CMDs among health and social care professionals in Sweden. Although there was no evidence of an overall difference in trends for the entire health care and social services sectors, there were variations in incidence rates for both outcome measures across specific occupational subgroups. Individuals working in medical laboratories and hospitals exhibited an increase in antidepressant prescription rates during the pandemic. Antidepressant prescription rates were increased particularly for younger individuals working in hospitals as well as in primary and dental care, and for women and those with a university or college degree working in medical laboratories during the pandemic compared with the pre-pandemic period.

The COVID-19 pandemic has been frequently discussed as posing a substantial burden on the mental health of people working in the health care and social care sectors because these were working on the frontline. Whereas studies have shown an increased short-term impact of the pandemic on the mental health of health personnel in Europe,^5,8^ studies assessing the long-term impact of the pandemic mostly suggest a stabilization of distress.^26,27^ Only 1 study suggested a persistence of symptoms of mental ill-health in health care individuals.^28^ Our investigation suggests that, in the Swedish context, the pandemic did not substantially worsen the overall long-term mental well-being of people working in the health care or social sector as expressed by SA or a prescription of antidepressants.

In line with previous studies, we observed variations in the different occupational groups, with certain groups being more affected than others.^9,13,28,29^ Specifically, individuals working in medical laboratories exhibited higher incidence rates for antidepressant prescription after the onset of the pandemic. The sudden increase in demand for diagnostic tests of a novel virus confronted this occupational group with more substantial change in working conditions and a steeply increased workload compared with other occupational groups. The current situation might have been further exacerbated by staff shortages, supply chain issues, and constant adaptations to the work environment.^30^ We found that within this group, women and people with a higher education were particularly affected, aligning with previous findings that women might be at greater risk of some CMDs, but might also be more likely to seek mental health support compared with men.^15,17^ In Sweden, the implementation of mass testing proved to be complex, involving a lot of issues, thus causing distress for more highly educated people, such as individuals working in managerial positions within laboratories.^31,32^

In contrast, individuals working in ambulance transports seemed to experience less challenge to their mental health, exhibiting lower IRs for antidepressant prescription after the onset of the pandemic. This finding contradicts 2 studies in which paramedics experienced higher levels of mental ill-health, with the assumption that their position as first responders was one of the possible reasons.^9,28^ In Sweden, efforts to prevent health care system overload and the transition of many health care services to telemedicine may have contributed to the reduced workload in ambulance services.^33^ This change in workload for some occupational groups, however, might only be temporary and a higher workload after the pandemic is expected, which might lead to increased work strain and chronic stress in occupational groups that were not at initial risk of mental ill-health during the pandemic.

Regarding absenteeism due to mental disorders, no discernible impact of the pandemic could be observed in our analyses. Health care professionals may adhere to a strong work ethic and morale, postponing sick leave and prioritizing support for colleagues and patients over their own well-being. Concepts, such as presenteeism, where individuals remain at their workplace regardless of their own well-being, are frequently discussed in the context of health care.^5,34^ In part our findings might be explained by increased sickness presenteeism (ie, employees who go to work despite being ill). Another explanation of our findings might be a normalization of feelings of exhaustion or psychological distress by health care and social workers who are working in an environment with an already high workload and pressure.^29^ Furthermore, in our study, we did not observe any increase in antidepressant prescription for the whole study population during the pandemic compared with pre-pandemic, which might further support an increase in stress-related disorders not requiring medication or people delaying seeking medical help due to the consequences of the pandemic on the health care system and people being encouraged to see a doctor only if really necessary.^35^

Before the onset of the pandemic, levels of SA >90 days due to CMDs and IRs for antidepressant prescription were already at a high level among health care professionals. For some occupational groups, such as those working in ambulatory settings, an increase in mental health challenges since the beginning of the study observation period (ie, before the start of the pandemic) could be observed. These findings underline the pre-existing pressure within the health care sector, often characterized by rising demands and coping with resource or staff shortages.^36^ It is likely that continuous increased work strain might inevitably lead to mental ill-health in the long term, which is why long-term mental health outcomes should be monitored in the future. In addition to monitoring, multi-component workplace interventions are needed at the organizational level to create a workplace that increases well-being—from adjusting the workload or establishing supportive leadership, to offering individual counseling services.^37,38^

### 4.1. Strengths and limitations

In our study we employed a population-wide design with objective measures derived from high-quality registers to longitudinally assess mental health outcomes, thus mitigating potential biases associated with self-reported measures. Furthermore, the population-based study design adopted for this study offers guarantees of representativeness and generalizability. Another strength is that the register-based nature of our study population precludes loss to follow-up. Still, there are some limitations. Both outcomes of our study were dependent on help-seeking behavior and if or how often a person used health care services at all, as well as how accessible those services were. The pandemic may have led to a reduced utilization of services due to recommendations to stay at home, and the widespread adoption of telemedicine, encouraging individuals to consult a doctor only when necessary to prevent health care system overload.^35^ It is likely, however, that these changes would only have impacted the IRs in the short-term, that is, in the early stage of the pandemic, and not have influenced our findings extending longer into the course of the pandemic. Second, for SA >90 days due to CMDs only the initial diagnosis was assessed for the SA spell, and this might have changed in the course of the SA period. Also, for antidepressant prescription no information on the indication of the medication was available, as antidepressants could also have been prescribed due to back pain or other musculoskeletal diagnoses.^39^ Additionally, the selection of the cut-off point was informed by the World Health Organization’s declaration of COVID-19 as a pandemic, rendering it non-deterministic.^25^ The virus had already spread before this point, potentially influencing working conditions. Nevertheless, due to the long-term nature of the measurement of our outcomes, it is unlikely that this could have significantly affected our findings. Lastly, our study pertains specifically to within-country trends in SA >90 days due to CMDs and antidepressant prescription, therefore the broader Northern European context needs to be considered. In comparison with other Western regions, Sweden has relatively high rates of chronic depression, which may influence baseline levels of CMD-related SA and help-seeking behavior.^40^ In addition, during the COVID-19 pandemic, Sweden had less strict measures for containing the pandemic and might have a different health care and social insurance system than other countries, which might have affected how mental health problems were translated into SA. Although cross-national comparisons were beyond the study’s scope, these regional characteristics provide important context for interpreting our findings.

## 5. Conclusions

Although the overall impact of the COVID-19 pandemic on long-term mental health was not substantial for most occupational groups in health care and social services in Sweden, staff in certain sectors such as medical laboratories and hospitals appeared more vulnerable. Within the specific occupational branches, a variability in sociodemographic characteristics could be observed, and individuals such as young people or women and those with a higher education were more affected in terms of increased incidence of antidepressant prescription. These findings emphasize the importance of considering subgroup variations in mental health outcomes within the health care profession during future crises.

## Supplementary Material

Web_Material_uiaf067

## Data Availability

The data used in this study cannot be made publicly available due to privacy regulations. According to the General Data Protection Regulation, the Swedish law SFS 2018:218, the Swedish Data Protection Act, the Swedish Ethical Review Act, and the Public Access to Information and Secrecy Act, these types of sensitive data can only be made available for specific purposes, including research, that meet the criteria for access to this sort of sensitive and confidential data as determined by a legal review. Readers may contact Professor Kristina Alexanderson (kristina.alexanderson@ki.se) regarding the data.
