# Supplementary material for: Sickness absence due to common mental disorders and antidepressant prescription among health and social care workers during compared with before the COVID-19 pandemic: a nationwide register study of the Swedish population
Source: J Occup Health. 2025 Nov 25;68(1):uiaf067. doi: 10.1093/joccuh/uiaf067 (PMC13043154; doi:10.1093/joccuh/uiaf067)
Supplement: Web_Material_uiaf067 [file web_material_uiaf067.docx]

**Supplementary material**

**Supplementary Table 1.** The occupational groups based on the SNI 2007 industry code grouping as well as the categories defined for the study

| **Code** | **Level 3 code** | **Level 4 code** | **Level 5 code** | **Category used in study** |
| --- | --- | --- | --- | --- |
| 86Q | (861) Hospital activities | (8610) Hospital activities | (86101) Hospital primary health activities | Hospital |
| 86Q | (861) Hospital activities | (8610) Hospital activities | (86102) Specialised hospital somatic activities | Hospital |
| 86Q | (861) Hospital activities | (8610) Hospital activities | (86103) Specialised hospital psychiatric activities | Hospital |
| 86Q | (862) Medical and dental practice activities | (8621) General medical practice activities | (86211) General primary medical practice activities | Primary care and dental |
| 86Q | (862) Medical and dental practice activities | (8621) General medical practice activities | (86212) Other general medical practice activities | Primary care and dental |
| 86Q | (862) Medical and dental practice activities | (8622) Specialist medical practice activities | (86221) Specialist medical practice activities, at hospitals | Hospital |
| 86Q | (862) Medical and dental practice activities | (8622) Specialist medical practice activities | (86222) Specialist medical practice activities, not at hospitals | Primary care and dental |
| 86Q | (862) Medical and dental practice activities | (8623) Dental practice activities | (86230) Dental practice activities | Primary care and dental |
| 86Q | (869) Other human health activities | (8690) Other human health activities | (86901) Activities of medical laboratories etc. | Medical laboratories |
| 86Q | (869) Other human health activities | (8690) Other human health activities | (86902) Ambulance transports and ambulance health care activities | Ambulance transports |
| 86Q | (869) Other human health activities | (8690) Other human health activities | (86903) Primary health activities, not physicians | Primary care, not physicians |
| 86Q | (869) Other human health activities | (8690) Other human health activities | (86904) Activities of dental hygienists | Primary care, not physicians |
| 86Q | (869) Other human health activities | (8690) Other human health activities | (86905) Activities of physiotherapists etc. | Primary care, not physicians |
| 86Q | (869) Other human health activities | (8690) Other human health activities | (86909) Other human health activities n.e.c. | Primary care, not physicians |
| 87Q | (871) Residential nursing care activities | (8710) Residential nursing care activities | (87100) Residential nursing care activities | Residential care activities, except elderly care |
| 87Q | (872) Residential care activities for mental retardation, mental health and substance abuse | (8720) Residential care activities for mental retardation, mental health and substance abuse | (87201) Care in special forms of accommodation for persons with mental retardation and mental disability | Residential care activities, except elderly care |
| 87Q | (872) Residential care activities for mental retardation, mental health and substance abuse | (8720) Residential care activities for mental retardation, mental health and substance abuse | (87202) Care in special forms of accommodation for children and young people with substance abuse problems | Residential care activities, except elderly care |
| 87Q | (872) Residential care activities for mental retardation, mental health and substance abuse | (8720) Residential care activities for mental retardation, mental health and substance abuse | (87203) Care in special forms of accommodation for adults with substance abuse problems | Residential care activities, except elderly care |
| 87Q | (873) Residential care activities for the elderly and disabled | (8730) Residential care activities for the elderly and disabled | (87301) Care in special forms of accommodation for the elderly | Elderly care |
| 87Q | (873) Residential care activities for the elderly and disabled | (8730) Residential care activities for the elderly and disabled | (87302) Care in special forms of accommodation for disabled persons | Residential care activities, except elderly care |
| 87Q | (879) Other residential care activities | (8790) Other residential care activities | (87901) Twenty-four hours care with accommodation for children and young people with social problems | Residential care activities, except elderly care |
| 87Q | (879) Other residential care activities | (8790) Other residential care activities | (87902) Care with accommodation for adults n.e.c. | Residential care activities, except elderly care |
| 88Q | (881) Social work activities without accommodation for the elderly and disabled | (8810) Social work activities without accommodation for the elderly and disabled | (88101) Social work activities without accommodation for the elderly | Social work |
| 88Q | (881) Social work activities without accommodation for the elderly and disabled | (8810) Social work activities without accommodation for the elderly and disabled | (88102) Social work activities without accommodation for disabled persons | Social work |
| 88Q | (889) Other social work activities without accommodation | (8891) Child day-care activities | (88910) Child day-care activities | Social work |
| 88Q | (889) Other social work activities without accommodation | (8899) Other social work activities without accommodation n.e.c. | (88991) Social work activities for children and young people with social problems | Social work |
| 88Q | (889) Other social work activities without accommodation | (8899) Other social work activities without accommodation n.e.c. | (88992) Day-care activities for adults with substance abuse problems | Social work |
| 88Q | (889) Other social work activities without accommodation | (8899) Other social work activities without accommodation n.e.c. | (88993) Social work activities without accommodation for adults n.e.c. | Social work |
| 88Q | (889) Other social work activities without accommodation | (8899) Other social work activities without accommodation n.e.c. | (88994) Humanitarian relief activities | Social work |
| 88Q | (889) Other social work activities without accommodation | (8899) Other social work activities without accommodation n.e.c. | (88995) Operation of refugee camps | Social work |

**Supplementary Figure 1.** Incidence rates (IR) of **anti-depressant prescription** per 1,000 person-years for different occupational groups in the time pre-pandemic (i.e., preceding the start of the COVID-19 pandemic, starting with the first quartile of 2018) and after the onset of the pandemic (starting from the second quartile of 2020 until the fourth quartile of 2021), **stratified by age**, adjusted for seasonality

**Supplementary Table 2.** Change in incidence rates (IR) and 95% Confidence Intervals (CI) and Slope incidence rate ratios (IRR) of **antidepressant prescription** per 1,000 person-years for different occupational groups in the time pre-pandemic (i.e., preceding the start of the COVID-19 pandemic, starting with the first quartile of 2018) and after the onset of the pandemic (starting from the second quartile of 2020 until the fourth quartile of 2021), **stratified by age**, adjusted for seasonality

|  |  |  |  |  |  |  |
| --- | --- | --- | --- | --- | --- | --- |
|  | **Before COVID-19 pandemic** | | **During COVID-19 pandemic** | | **During vs before COVID-19 pandemic** | |
| **Occupational groups** | **Slope IR (95% CI)** | **P-value** | **Slope IR (95% CI)** | **P-value** | **Slope IRR (95% CI)** | **P-value** |
| **All occupations** |  |  |  |  |  |  |
| 19-25 | 0.987 (0.973-1.000) | 0.049 | 1.012 (0.995-1.030) | 0.176 | 1.026 (0.998-1.055) | 0.070 |
| 26-35 | 0.999 (0.987-1.011) | 0.912 | 1.003 (0.984-1.023) | 0.742 | 1.004 (0.976-1.032) | 0.783 |
| 36-45 | 1.002 (0.985-1.019) | 0.837 | 1.005 (0.984-1.027) | 0.639 | 1.003 (0.970-1.038) | 0.844 |
| 46-55 | 1.007 (0.988-1.027) | 0.488 | 1.010 (0.986-1.034) | 0.440 | 1.003 (0.965-1.042) | 0.894 |
| 56-64 | 1.005 (0.991-1.020) | 0.475 | 1.016 (0.997-1.035) | 0.102 | 1.010 (0.981-1.041) | 0.488 |
| **Hospital** |  |  |  |  |  |  |
| 19-25 | 0.985 (0.974-0.996) | 0.010 | 1.038 (1.023-1.055) | <0.001 | 1.054 (1.029-1.080) | <0.001 |
| 26-35 | 0.994 (0.981-1.007) | 0.375 | 1.018 (0.997-1.039) | 0.088 | 1.024 (0.994-1.056) | 0.122 |
| 36-45 | 1.007 (0.991-1.024) | 0.367 | 1.009 (0.984-1.034) | 0.486 | 1.001 (0.966-1.038) | 0.936 |
| 46-55 | 1.009 (0.989-1.029) | 0.399 | 1.012 (0.991-1.033) | 0.258 | 1.003 (0.968-1.040) | 0.855 |
| 56-64 | 1.004 (0.982-1.027) | 0.724 | 1.023 (0.996-1.051) | 0.094 | 1.019 (0.975-1.065) | 0.398 |
| **Primary care and dental** |  |  |  |  |  |  |
| 19-25 | 0.978 (0.950-1.006) | 0.129 | 1.049 (1.020-1.079) | 0.001 | 1.073 (1.017-1.131) | 0.010 |
| 26-35 | 0.997 (0.976-1.018) | 0.761 | 1.016 (0.978-1.056) | 0.412 | 1.019 (0.966-1.076) | 0.487 |
| 36-45 | 1.012 (0.992-1.033) | 0.242 | 1.006 (0.984-1.029) | 0.601 | 0.994 (0.957-1.032) | 0.745 |
| 46-55 | 1.008 (0.985-1.031) | 0.498 | 1.018 (0.995-1.042) | 0.119 | 1.010 (0.972-1.051) | 0.603 |
| 56-64 | 1.007 (0.986-1.029) | 0.492 | 1.027 (1.006-1.049) | 0.013 | 1.019 (0.983-1.057) | 0.294 |
| **Medical laboratories** |  |  |  |  |  |  |
| 19-25 | 0.959 (0.750-1.226) | 0.736 | 1.071 (0.805-1.425) | 0.637 | 1.117 (0.710-1.759) | 0.632 |
| 26-35 | 0.987 (0.882-1.105) | 0.822 | 1.083 (1.021-1.148) | 0.008 | 1.097 (0.937-1.284) | 0.249 |
| 36-45 | 0.957 (0.895-1.022) | 0.191 | 1.050 (0.981-1.125) | 0.160 | 1.098 (0.979-1.231) | 0.109 |
| 46-55 | 0.938 (0.880-0.999) | 0.047 | 1.017 (0.935-1.106) | 0.691 | 1.085 (0.940-1.251) | 0.266 |
| 56-64 | 0.956 (0.901-1.015) | 0.140 | 1.041 (0.958-1.132) | 0.341 | 1.089 (0.964-1.230) | 0.169 |
| **Ambulance transports** |  |  |  |  |  |  |
| 19-25 | 0.937 (0.717-1.226) | 0.636 | 1.212 (0.902-1.628) | 0.203 | 1.293 (0.790-2.116) | 0.307 |
| 26-35 | 1.021 (0.963-1.083) | 0.482 | 0.991 (0.910-1.080) | 0.838 | 0.970 (0.856-1.100) | 0.639 |
| 36-45 | 1.121 (1.009-1.245) | 0.033 | 0.961 (0.884-1.044) | 0.341 | 0.857 (0.731-1.004) | 0.056 |
| 46-55 | 1.052 (1.001-1.104) | 0.044 | 0.955 (0.887-1.027) | 0.214 | 0.908 (0.814-1.013) | 0.084 |
| 56-64 | 0.892 (0.838-0.949) | <0.001 | 1.084 (0.993-1.184) | 0.071 | 1.215 (1.064-1.388) | 0.004 |
| **Primary care, not physicians** |  |  |  |  |  |  |
| 19-25 | 1.016 (0.940-1.098) | 0.691 | 1.057 (0.955-1.170) | 0.282 | 1.041 (0.884-1.225) | 0.633 |
| 26-35 | 1.000 (0.972-1.030) | 0.979 | 1.007 (0.977-1.038) | 0.640 | 1.007 (0.953-1.063) | 0.807 |
| 36-45 | 0.999 (0.971-1.027) | 0.925 | 0.991 (0.963-1.019) | 0.528 | 0.992 (0.946-1.041) | 0.752 |
| 46-55 | 0.980 (0.942-1.020) | 0.319 | 1.000 (0.955-1.047) | 0.994 | 1.021 (0.941-1.107) | 0.622 |
| 56-64 | 1.011 (0.978-1.044) | 0.527 | 0.993 (0.942-1.047) | 0.796 | 0.983 (0.914-1.056) | 0.633 |
| **Residential care activities, except elderly care** |  |  |  |  |  |  |
| 19-25 | 0.996 (0.961-1.032) | 0.811 | 1.014 (0.960-1.072) | 0.612 | 1.019 (0.938-1.107) | 0.660 |
| 26-35 | 1.015 (0.998-1.032) | 0.077 | 0.991 (0.977-1.004) | 0.163 | 0.976 (0.950-1.002) | 0.073 |
| 36-45 | 1.003 (0.978-1.029) | 0.804 | 1.006 (0.982-1.031) | 0.634 | 1.003 (0.957-1.051) | 0.909 |
| 46-55 | 0.997 (0.975-1.020) | 0.786 | 1.018 (0.996-1.040) | 0.111 | 1.021 (0.982-1.062) | 0.300 |
| 56-64 | 1.007 (0.989-1.024) | 0.458 | 1.007 (0.988-1.028) | 0.466 | 1.001 (0.969-1.034) | 0.959 |
| **Elderly care** |  |  |  |  |  |  |
| 19-25 | 0.983 (0.960-1.007) | 0.167 | 1.013 (0.984-1.044) | 0.370 | 1.031 (0.979-1.085) | 0.248 |
| 26-35 | 0.995 (0.979-1.012) | 0.581 | 1.005 (0.987-1.023) | 0.607 | 1.009 (0.979-1.040) | 0.548 |
| 36-45 | 1.006 (0.986-1.026) | 0.554 | 1.003 (0.979-1.029) | 0.792 | 0.997 (0.959-1.038) | 0.897 |
| 46-55 | 1.014 (0.994-1.034) | 0.173 | 0.999 (0.970-1.030) | 0.967 | 0.986 (0.944-1.030) | 0.522 |
| 56-64 | 1.015 (1.002-1.029) | 0.028 | 1.006 (0.992-1.020) | 0.424 | 0.991 (0.968-1.014) | 0.444 |
| **Social work** |  |  |  |  |  |  |
| 19-25 | 0.987 (0.974-1.000) | 0.046 | 1.001 (0.984-1.019) | 0.900 | 1.015 (0.988-1.042) | 0.295 |
| 26-35 | 1.003 (0.990-1.016) | 0.669 | 0.994 (0.975-1.014) | 0.561 | 0.991 (0.964-1.020) | 0.549 |
| 36-45 | 0.991 (0.973-1.010) | 0.369 | 1.003 (0.984-1.022) | 0.751 | 1.012 (0.979-1.045) | 0.478 |
| 46-55 | 1.010 (0.989-1.033) | 0.351 | 1.011 (0.980-1.043) | 0.490 | 1.001 (0.953-1.050) | 0.982 |
| 56-64 | 1.001 (0.979-1.023) | 0.945 | 1.018 (0.996-1.041) | 0.110 | 1.018 (0.978-1.059) | 0.395 |
|  |  |  |  |  |  |  |

**Supplementary Figure 2.** Incidence rates of **antidepressant prescription** per 1,000 person-years for different occupational groups in the time pre-pandemic (i.e., preceding the start of the COVID-19 pandemic, starting with the first quartile of 2018) and after the onset of the pandemic (starting from the second quartile of 2020 until the fourth quartile of 2021), **stratified by sex**, adjusted for seasonality

**Supplementary Table 3.** Change in incidence rates (IR) and 95% Confidence Intervals (CI) and Slope incidence rate ratios (IRR) of **antidepressant prescription** per 1,000 person-years for different occupational groups in the time pre-pandemic (i.e., preceding the start of the COVID-19 pandemic, starting with the first quartile of 2018) and after the onset of the pandemic (starting from the second quartile of 2020 until the fourth quartile of 2021), **stratified by sex**, adjusted for seasonality

|  |  |  |  |  |  |  |
| --- | --- | --- | --- | --- | --- | --- |
|  | **Before COVID-19 pandemic** | | **During COVID-19 pandemic** | | **During vs before COVID-19 pandemic** | |
| **Occupational groups** | **Slope IR (95% CI)** | **P-value** | **Slope IR (95% CI)** | **P-value** | **Slope IRR (95% CI)** | **P-value** |
| **All occupations** |  |  |  |  |  |  |
| Women | 1.001 (0.987-1.015) | 0.887 | 1.010 (0.991-1.030) | 0.298 | 1.009 (0.980-1.040) | 0.533 |
| Men | 1.004 (0.987-1.021) | 0.643 | 1.000 (0.976-1.024) | 0.980 | 0.996 (0.959-1.033) | 0.822 |
| **Hospital** |  |  |  |  |  |  |
| Women | 1.003 (0.989-1.018) | 0.630 | 1.017 (0.995-1.040) | 0.129 | 1.014 (0.982-1.046) | 0.396 |
| Men | 0.996 (0.979-1.013) | 0.659 | 1.013 (0.998-1.030) | 0.096 | 1.017 (0.988-1.047) | 0.247 |
| **Primary care and dental** |  |  |  |  |  |  |
| Women | 1.005 (0.987-1.023) | 0.615 | 1.020 (0.996-1.044) | 0.106 | 1.015 (0.978-1.054) | 0.427 |
| Men | 1.021 (0.993-1.051) | 0.144 | 1.003 (0.973-1.034) | 0.836 | 0.982 (0.932-1.035) | 0.501 |
| **Medical laboratories** |  |  |  |  |  |  |
| Women | 0.953 (0.936-0.971) | <0.001 | 1.057 (1.037-1.076) | <0.001 | 1.109 (1.072-1.147) | <0.001 |
| Men | 1.005 (0.876-1.154) | 0.941 | 1.034 (0.874-1.224) | 0.696 | 1.029 (0.766-1.381) | 0.851 |
| **Ambulance transports** |  |  |  |  |  |  |
| Women | 1.060 (1.007-1.116) | 0.026 | 0.954 (0.875-1.040) | 0.282 | 0.899 (0.797-1.015) | 0.084 |
| Men | 1.005 (0.963-1.049) | 0.807 | 1.026 (0.939-1.120) | 0.573 | 1.020 (0.906-1.149) | 0.742 |
| **Primary care, not physicians** |  |  |  |  |  |  |
| Women | 0.996 (0.977-1.016) | 0.692 | 1.005 (0.978-1.033) | 0.721 | 1.009 (0.968-1.052) | 0.673 |
| Men | 1.006 (0.969-1.044) | 0.766 | 0.970 (0.925-1.018) | 0.218 | 0.965 (0.895-1.040) | 0.353 |
| **Residential care activities, except elderly care** |  |  |  |  |  |  |
| Women | 1.001 (0.986-1.015) | 0.939 | 1.008 (0.994-1.021) | 0.265 | 1.007 (0.985-1.030) | 0.543 |
| Men | 1.012 (0.977-1.048) | 0.512 | 0.999 (0.960-1.040) | 0.956 | 0.987 (0.920-1.059) | 0.719 |
| **Elderly care** |  |  |  |  |  |  |
| Women | 1.004 (0.989-1.020) | 0.593 | 1.006 (0.985-1.028) | 0.592 | 1.002 (0.969-1.035) | 0.926 |
| Men | 1.007 (0.989-1.026) | 0.428 | 0.994 (0.973-1.015) | 0.561 | 0.987 (0.952-1.023) | 0.460 |
| **Social work** |  |  |  |  |  |  |
| Women | 0.999 (0.984-1.013) | 0.850 | 1.007 (0.989-1.026) | 0.454 | 1.008 (0.980-1.038) | 0.567 |
| Men | 1.003 (0.985-1.021) | 0.757 | 0.994 (0.962-1.026) | 0.690 | 0.991 (0.947-1.037) | 0.688 |
|  |  |  |  |  |  |  |

**Supplementary Figure 3.** Incidence rates of **antidepressant prescription** per 1,000 person-years for different occupational groups in the time pre-pandemic (i.e., preceding the start of the COVID-19 pandemic, starting with the first quartile of 2018) and after the onset of the pandemic (starting from the second quartile of 2020 until the fourth quartile of 2021), **stratified by education**, adjusted for seasonality

**Supplementary Table 4.** Change in incidence rates (IR) and 95% Confidence Intervals (CI) and Slope incidence rate ratios (IRR) of **antidepressant prescription** per 1,000 person-years for different occupational groups in the time pre-pandemic (i.e., preceding the start of the COVID-19 pandemic, starting with the first quartile of 2018) and after the onset of the pandemic (starting from the second quartile of 2020 until the fourth quartile of 2021), **stratified by education**, adjusted for seasonality

|  |  |  |  |  |  |  | |
| --- | --- | --- | --- | --- | --- | --- | --- |
|  | **Before COVID-19 pandemic** | | **During COVID-19 pandemic** | | **During vs before COVID-19 pandemic** | |  |
| **Occupational groups** | **Slope IR (95% CI)** | **P-value** | **Slope IR (95% CI)** | **P-value** | **Slope IRR (95% CI)** | **P-value** | |
| **All occupations** |  |  |  |  |  |  | |
| Elementary (≤9 years)/High school (10-12 years) | 1.001 (0.987-1.015) | 0.928 | 1.007 (0.988-1.026) | 0.487 | 1.006 (0.977-1.036) | 0.683 | |
| University/college (>12 years) | 1.003 (0.988-1.018) | 0.683 | 1.011 (0.990-1.033) | 0.295 | 1.008 (0.977-1.041) | 0.620 | |
| **Hospital** |  |  |  |  |  |  | |
| Elementary (≤9 years)/High school (10-12 years) | 0.998 (0.983-1.013) | 0.816 | 1.017 (0.992-1.042) | 0.186 | 1.019 (0.984-1.055) | 0.298 | |
| University/college (>12 years) | 1.004 (0.989-1.020) | 0.586 | 1.017 (0.998-1.036) | 0.088 | 1.012 (0.982-1.044) | 0.431 | |
| **Primary care and dental** |  |  |  |  |  |  | |
| Elementary (≤9 years)/High school (10-12 years) | 1.017 (1.000-1.036) | 0.056 | 1.011 (0.982-1.040) | 0.461 | 0.993 (0.953-1.036) | 0.758 | |
| University/college (>12 years) | 1.004 (0.987-1.021) | 0.674 | 1.019 (0.998-1.041) | 0.080 | 1.016 (0.981-1.051) | 0.376 | |
| **Medical laboratories** |  |  |  |  |  |  | |
| Elementary (≤9 years)/High school (10-12 years) | 0.940 (0.893-0.990) | 0.019 | 1.019 (0.969-1.071) | 0.462 | 1.083 (0.990-1.186) | 0.083 | |
| University/college (>12 years) | 0.965 (0.935-0.995) | 0.023 | 1.061 (1.029-1.094) | <0.001 | 1.100 (1.041-1.161) | 0.001 | |
| **Ambulance transports** |  |  |  |  |  |  | |
| Elementary (≤9 years)/High school (10-12 years) | 0.957 (0.881-1.040) | 0.301 | 1.016 (0.922-1.120) | 0.748 | 1.062 (0.912-1.236) | 0.441 | |
| University/college (>12 years) | 1.069 (1.039-1.100) | <0.001 | 0.976 (0.936-1.017) | 0.239 | 0.913 (0.857-0.972) | 0.004 | |
| **Primary care, not physicians** |  |  |  |  |  |  | |
| Elementary (≤9 years)/High school (10-12 years) | 0.996 (0.970-1.023) | 0.787 | 1.033 (0.991-1.077) | 0.124 | 1.037 (0.974-1.104) | 0.259 | |
| University/college (>12 years) | 0.998 (0.981-1.016) | 0.836 | 0.990 (0.970-1.011) | 0.343 | 0.992 (0.961-1.024) | 0.616 | |
| **Residential care activities, except elderly care** |  |  |  |  |  |  | |
| Elementary (≤9 years)/High school (10-12 years) | 1.010 (0.994-1.026) | 0.238 | 1.000 (0.983-1.017) | 0.977 | 0.990 (0.962-1.019) | 0.505 | |
| University/college (>12 years) | 0.992 (0.970-1.014) | 0.482 | 1.017 (0.997-1.038) | 0.098 | 1.026 (0.987-1.066) | 0.200 | |
| **Elderly care** |  |  |  |  |  |  | |
| Elementary (≤9 years)/High school (10-12 years) | 1.002 (0.989-1.016) | 0.727 | 1.007 (0.987-1.026) | 0.507 | 1.004 (0.974-1.035) | 0.793 | |
| University/college (>12 years) | 1.012 (0.988-1.036) | 0.324 | 0.996 (0.967-1.026) | 0.783 | 0.984 (0.940-1.030) | 0.497 | |
| **Social work** |  |  |  |  |  |  | |
| Elementary (≤9 years)/High school (10-12 years) | 0.996 (0.981-1.012) | 0.614 | 1.005 (0.986-1.024) | 0.614 | 1.009 (0.979-1.040) | 0.566 | |
| University/college (>12 years) | 1.006 (0.991-1.021) | 0.437 | 1.003 (0.979-1.028) | 0.817 | 0.997 (0.962-1.033) | 0.869 | |
|  |  |  |  |  |  |  | |
